# Geometric error of cervical point A calculated through traditional reconstruction procedures for brachytherapy treatment

**DOI:** 10.1120/jacmp.v16i5.5558

**Published:** 2015-09-08

**Authors:** Liyun Chang, Sheng‐Yow Ho, Shyh‐An Yeh, Tsair‐Fwu Lee, Pang‐Yu Chen

**Affiliations:** ^1^ Department of Medical Imaging and Radiological Sciences I‐Shou University Kaohsiung Taiwan; ^2^ Department of Nursing Chang Jung Christian University Tainan Taiwan; ^3^ Department of Radiation Oncology Chi Mei Medical Center, Liouying Tainan Taiwan; ^4^ Department of Radiation Oncology E‐Da Hospital Kaohsiung Taiwan; ^5^ Medical Physics and Informatics Laboratory Department of Electronics Engineering, National Kaohsiung University of Applied Sciences Kaohsiung Taiwan; ^6^ Department of Radiation Oncology Sinlau Christian Hospital Tainan Taiwan

**Keywords:** brachytherapy, cervical cancer, point A, geometric error

## Abstract

Brachytherapy used in local cervical cancer is still widely based on 2D standard dose planning with the prescription to point A, which is invisible on imaging and located at a high‐dose gradient. In this study, the geometric location error of point A was investigated. It is traditionally reconstructed in the treatment planning system after carefully digitizing the point marks that were previously drawn on the orthogonal radiographs into the system. Two Cartesian coordinates of point A were established and compared. One was built up based on the geometric definition of point A and would be taken as the true coordinate, while the other was built up through traditional clinical treatment procedures and named as the practical coordinate. The orthogonal film reconstruction technique was used and the location error between the practical and the true coordinate introduced from the variations of, first, the angle between the tandem and the simulator gantry rotation axis, and second, the interval between the tandem flange and the simulator isocenter, was analyzed. The location error of point A was higher if the tandem was rotated away from the gantry rotation axis or if the location of the tandem flange was set away from the isocenter. If a tandem with a 30° curvature was rotated away from the gantry rotation axis 10° in the anterior–posterior (AP) view, and there was an 8.7 cm interval between the flange and the isocenter, the location error of point A would be 3 mm without including other errors from simulator calibration, data input, patient setup, and movements. To reduce the location error of point A calculated for traditional reconstruction procedures, it is suggested to move the couch or patient to make the mid‐point of two points A near the isocenter and the tandem in the AP view parallel to the gantry rotation axis as much as possible.

PACS number: 87.55.km

## I. INTRODUCTION

Brachytherapy (BT) has been widely used for decades in the adjuvant treatment of cervical carcinoma.^1^, ^2^, ^3^, ^4^ For the curative treatment of all stages, BT truly plays an essential role, giving the patient a needed boost dose.^3^, ^4^, ^5^, ^6^, ^7^ Through delivering a substantially high dose to the tumor in the central pelvis, while sparing the nearby organs at risk due to the rapid dose falloff,^(8)^ BT leads to an improvement in the patient survival rate with a decrease in the patient recurrence rate.^9^, ^10^, ^11^, ^12^ Hence, there is no doubt that the curative potential of radiation therapy in the management of cervical cancer was proved to be greatly enhanced by the use of intracavitary BT.^9^, ^13^, ^14^, ^15^

Throughout decades of clinical practice and as a result of the abundant experience accumulated by radiation oncologists, delivery of a certain dose to point A is still a commonly used prescription for cervical cancer BT.^16^, ^17^, ^18^ Besides delivering the desired dose to point A, the associated isodose curves are also preferred to be a pear shape with the widest part near the cervix.^(19)^ Several definitions have been used historically to define the location of point A in terms of its location along the direction of the tandem (intrauterine probes). In the earliest Manchester system,^(20)^ point A was defined at “2 cm lateral to the central canal of the uterus, and 2 cm up from the mucous membrane of the lateral fornix in the axis of the uterus”. In 1953, the definition of point A was modified as a point 2 cm superior to the external cervical os and 2 cm lateral to the cervical canal.^(21)^ The modified definition is still referenced now in standard medical physics textbooks.^(22)^ In the Madison system (developed by the University of Wisconsin), the reference point M was used instead of point A and was defined as “2 cm lateral to the center of the uterine canal and 2 cm cephalad from a line joining the center dwell position of the vaginal colpostat sources”.^(23)^ Recently, the earliest definition of point A has been readopted with some adjustments by the American Brachytherapy Society (ABS)^24^, ^25^, ^26^ and European Society for Therapeutic Radiation Oncology (ESTRO).^7^, ^27^

The AAPM TG 56 has recommended that “a major function of the physicist is to maintain consistency between past and current practice with respect to applicator dosimetric characteristics and calculation of prescription and treatment constraining parameters such as reference point doses (rectal dose, point A dose...)”.^(28)^ A project of EQUAL‐ESTRO reported that “a 0.5 mm deviation in distance relative to a treatment distance of 20 mm in brachytherapy means a 5% variation in dose delivery”.^(29)^ For low‐dose‐rate brachytherapy, Zhang et al.^(30)^ demonstrated that a 9 mm shift in point A can cause a 14% dose rate difference. Another ESTRO study demonstrated the high‐dose gradient around point A, in that “the dose along an axis perpendicular to the intrauterine source at the level of point A decreases from approximately 200% to 100% of the dose to point A when going from 10 to 20 mm from the source, whereas the dose decreases from 100% to approximately 60% from 20 to 30 mm”.^(27)^ It is important for physicists to get the correct location of point A for each individual treatment, since a slight variation of its location can result in significant dose variation.^16^, ^30^, ^31^

The position of point A is still widely calculated based on 2D X‐ray imaging^(32)^ and is generally reconstructed through the point marks predrawn on the orthogonal radiographs.^33^, ^34^, ^35^ However, as indicated by Bentel,^(19)^ “Although point A is defined in relation to important anatomic structures, these cannot be visualized on a radiograph.” According to the geometric definition of point A, its location is not easily determined exactly on a radiograph, due to the unknown magnification of it on film. Therefore, the goal of our study is to investigate the geometric location error of point A, which is traditionally reconstructed from the predrawn point marks. The dependence of the error will be analyzed on the angle between the tandem and the simulator gantry rotation axis, and the interval between the tandem flange and the simulator isocenter.

## II. MATERIALS AND METHODS

**Figure 1:**
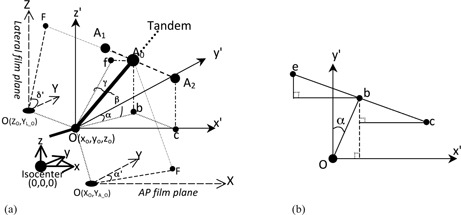
The geometric illustration of the tandem and point A in space with the coordinates on the AP and lateral films (Fig. 2 and Fig 3): (a) illustration of point A in space; (b) the projection of point A on the $x^{\prime }-y^{\prime }$ plane. The point O is represented as the position of os (or the flange) with the coordinate ($x_{o},y_{o},z_{o}$); $x^{\prime },y^{\prime }-$, and $z^{\prime }$‐axes pass through the point O, directed as the x−,y−‐, and z‐axis, respectively; F is the projection of $A_{0}$ on the AP and lateral films; the coordinates of the projections of O on the AP and lateral films are ($X_{O},Y_{A\_O}$) and ($Z_{O},Y_{L\_O}$) respectively. $A_{0}$ is the midpoint between $A_{1}$ and $A_{2}$ and ${\text{OA}}_{0}$ is 2 cm along the tandem; α is the angle between the y‐axis and the tandem $\bar{{\text{OT}}_{0}}$ projection on the $x^{\prime },y^{\prime }$ plane; β is the angle of $\bar{{\text{OT}}_{0}}$ with the $x^{\prime },y^{\prime }$ plane; e, b, and c are the projections of the points $A_{1},A_{0}$, and $A_{2}$ on the $x^{\prime },y^{\prime }$ plane, respectively.

In our department, five or six fractionated boost doses are given to the cervical cancer patient through an Ir‐192 HDR system. Before beginning BT treatment for cervical cancer, the patient is placed in a supine position on a movable homemade couch with her feet facing the gantry of a Toshiba DC50N simulator (Tokyo, Japan), and then the orthogonal X‐ray images are taken for film reconstruction. To calculate point A, we first need to define a Cartesian coordinate in the patient with the origin at the simulator isocenter, in which the z‐axis parallels gravity but in the opposite direction, the y‐axis parallels the gantry rotation axis but is directed away from the gantry, and the x‐axis points to the left of the patient. Another three axes, $x^{\prime },y^{\prime }$, and $z^{\prime }$, are defined with the same directions as the x−,y−, and z‐axis, respectively (Fig. 1), and pass through a point O, which has the coordinate ($x_{o},y_{o},z_{o}$).

In our clinic, the definition of the two points A was based on the modified Manchester system and is represented as $A_{1}$ and $A_{2}$, the left and right point A on the anterior–posterior (AP) film image (head up), respectively, each located 2 cm superior to the external cervical os and 2 cm right and left lateral to the patient's cervical canal. In the traditional orthogonal film reconstruction, point A would be delineated starting from the radiopaque flange of the tandem that should be adjacent to the cervical os. The point O is set at the location of the flange (Fig. 1), which is also the assumed position of the cervical os. For the calculation in the other system, point O can be located at a different position based on the definition of point A. For instance, in the Madison system, point O could be at point M, which is defined at the center dwell position of the ovoid colpostat sources.^(23^) For the ABS and ESTRO system, O could stand for the point located at the tandem above the intersection of the tandem and the line connecting the two ovoid mid‐dwell positions by the length of the ovoid radius along the tandem.

During treatment, the patient lies flat on the couch, so that the line connecting the two points A can be taken as parallel to the x‐y plane (or the $x^{\prime }-y^{\prime }$ plane). The tandem is defined as β degree titled from the $x^{\prime }-y^{\prime }$ plane (usually this is the curvature angle of the probe). The angle between the y‐axis and the projection of the tandem at the $x^{\prime }-y^{\prime }$ plane is defined as α degree (Fig. 1). To get the location of point A, the coordinate of point O, ($x_{o},y_{o},z_{o}$), is needed and can be calculated through the orthogonal images (please refer to Appendix A).

### A. The calculation of the true coordinate of point A

According to Fig. 1(a), the z coordinates of $A_{1}$ and $A_{2},z_{A_{1}}$ and $z_{A_{2}}$, respectively, could be written as

(1) $$z_{A1}=z_{A2}=\bar{OA_{0}}\cdot \text{sin}\beta +z_{o}=2cm\cdot \text{sin}\beta +z_{o}$$

Through Fig. 1(b), the x and y coordinates of $A_{1}$ and $A_{2},A_{2},z_{A_{1}}$, and $z_{A_{2}}$, respectively, could be written in order:

(2) $$x_{A_{1}}=2cm\cdot \text{cos}\beta \cdot \text{sin}\alpha -2cm\cdot \text{cos}\alpha +x_{o}$$

(3) $$x_{A_{2}}=2cm\cdot \text{cos}\beta \cdot \text{sin}\alpha +2cm\cdot \text{cos}\alpha +x_{o}$$

(4) $$y_{A_{1}}=2cm\cdot \text{cos}\beta \cdot \text{cos}\alpha +2cm\cdot \text{sin}\alpha +y_{o}$$

(5) $$y_{A_{2}}=2cm\cdot \text{cos}\beta \cdot \text{cos}\alpha -2cm\cdot \text{sin}\alpha +y_{o}$$

**Figure 2:**
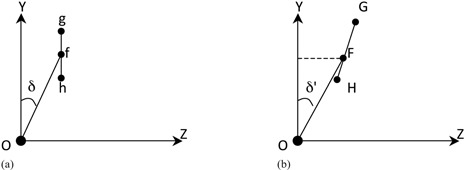
The projection of points $A_{0},A_{1}$, and $A_{2}$ on the film cassette plane (β=−90°) is represented as (a) point f, g, and h, respectively, by a parallel source (without magnification), and (b) points F, G, and H, respectively, by a point source. δ and $\delta^{\prime }$ is the angle between $\bar{Of}$ and the Y‐axis and the angle between $\bar{Of}$ and the Y‐axis, respectively. (a) parallel radiation source; (b) point radiation source.

To study the coordinate of point A on the lateral image, Fig. 2 illustrates the lateral view (gantry angle θ is −90°, as defined in the Appendix) of points $A_{0},A_{1}$, and $A_{2}$ at the film cassette plane with a) an imaginary parallel radiation source (no magnification), and b) a point radiation source. The parallel source introduced here would help in thinking of the point source, a more complicated situation. Capital X, Y, and Z were used to indicate the three axes of the Cartesian coordinate system on the radiograph.

The location of $A_{1}$ is farther away from the film cassette (θ=−90°) than that of $A_{2}$. For the parallel source, the two Z coordinates of g and h are equal and $\bar{fg}$ is equal to $\bar{fh}$. For the point source, due to the magnification, the Y and Z coordinate of point G should be larger than that of H. Compared to the $\bar{fg}$ in Fig. 2(a), $\bar{FG}$ is longer and rotated slightly clockwise in Fig. 2(b). According to (1), (2), (3), (4), (5) in the Appendix, the coordinates of F, G, and H, ($Z_{F},Y_{F}$), ($Z_{G},Y_{G}$), and ($Z_{H},Y_{H}$), respectively, can be written as

(6) $$Z_{F}=(2\text{sin}\beta +z_{o})\times SFD/(100+x_{o}+2cos\beta \text{sin}\alpha )$$

(7) $$Y_{F}=(2\text{cos}\beta \text{cos}\alpha +y_{o})\times SFD/(100+x_{o}+2\text{cos}\beta \text{sin}\alpha )$$

(8) $$Z_{G}=(2\text{sin}\beta +z_{o})\times SFD/(100+x_{o}+2\text{cos}\beta \text{sin}\alpha -2\text{cos}\alpha )$$

(9) $$Y_{G}=(2\text{cos}\beta \text{cos}\alpha +2\text{sin}\alpha +y_{o})\times SFD/(100+x_{o}+2\text{cos}\beta \text{sin}\alpha -2\text{cos}\alpha )$$

(10) $$Z_{H}=(2\text{sin}\beta +z_{o})\times SFD/(100+x_{o}+2\text{cos}\beta \text{sin}\alpha +2\text{cos}\alpha )$$

(11) $$Y_{H}=(2\text{cos}\beta \text{cos}\alpha -2\text{sin}\alpha +y_{o})\times SFD/(100+x_{o}+2\text{cos}\beta \text{sin}\alpha +2\text{cos}\alpha )$$

where *SFD* is the source–film distance. We then could see that the Z coordinate is not the same value for F, G, and H and the distance between and $\bar{\text{FG}}$ is not equal either. Through (6), (7) and Fig. 2(b), the $\delta^{\prime }$ can be calculated by:

(12) $$\text{tan}\delta^{\prime }=\frac{\left(2\text{sin}\beta +z_{o})/(100+x_{o}+2\text{cos}\beta \text{sin}\alpha )-z_{o}/(100+x_{o}\right)}{\left(2\text{cos}\beta \text{cos}\alpha +y_{o})/(100+x_{o}+2\text{cos}\beta \text{sin}\alpha )-y_{o}/(100+x_{o}\right)}$$

Similarly, the tangent of $\alpha^{\prime }$ (Fig. 1(a)) can be written as:

(13) $$\text{tan}\alpha^{\prime }=\frac{\left(2\text{cos}\beta \text{sin}\alpha +x_{o})/(100-z_{o}-2\text{sin}\beta )-x_{o}/(100-z_{o}\right)}{\left(2\text{cos}\beta \text{cos}\alpha +y_{o})/(100-z_{o}-2\text{sin}\beta )-y_{o}/(100-z_{o}\right)}$$

$\delta^{\prime }$ and $\alpha^{\prime }$ will be used in the next section.

### B. Calculation of the practical coordinate of point A

**Figure 3:**
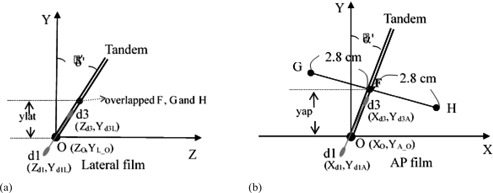
The relative positions between point O, F, G, H, projection of dummy d1 and d3 for the traditional method to mark point A on (a) lateral and (b) AP film. Source–film distance was set at 140 cm. $\delta^{\prime }$ and $\alpha^{\prime }$ is the angle between the tandem and the Y‐axis on the lateral and AP film, respectively.

The two points A were traditionally marked on the AP and lateral films before digitizing them into a treatment planning system to reconstruct their location in the 3D space. Two dummy seeds (d1 and d3 in Fig. 3) with 2 cm separation near the point O in the tandem generally would be used to mark the midpoint F of the point $A_{1}$ and $A_{2}$ (renamed as G and H, respectively, here) on the AP and lateral film, where the lower dummy seeds of the two (d1 in Fig. 3) is chosen as the one nearest the point O of all the dummy seeds. F then can be located and marked at the AP film, which is above the flange by the length of $\bar{d1d3}$, the distance measured by a ruler between the d1 and d3 on the AP film, along the tandem (Fig. 3). That is, $\bar{\text{OF}}$ is equal to $\bar{d1d3}$ on the film. F was also marked on the lateral film in the same way. Usually, for convenience, G and H are marked overlapping with F on the lateral film. Since our SFD is set at 140 cm, the object magnification on film would be taken as 1.4. On the AP film, G and H would be located at 2.8 cm away from the F, perpendicularly to the tandem (Fig. 3).

The dummy d1 and d3 (Fig. 3) were supposed to be d1 and d3 cm away from the point O along the tandem (above the point O is taken as positive), respectively. According to Fig. 1, the coordinate of dummy $d1,(x_{d1},y_{d1},z_{d1})$, and dummy $d3,(x_{d3},y_{d3},z_{d3})$, can be written as $\left(d_{1}\times \text{cos}\beta \text{sin}\alpha +x_{0},d_{1}\times \text{cos}\beta \text{cos}\alpha +y_{0},d_{1}\times \text{sin}\beta +z_{0}\right)$ and $\left(d_{3}\times \text{cos}\beta \text{sin}\alpha +x_{0},d_{3}\times \text{cos}\beta \text{cos}\alpha +y_{0},d_{3}\times \text{sin}\beta +z_{0}\right)$, respectively. According to (A1), (A2), (A3) with θ=−90°, the coordinates of dummy $d_{1}$ on the AP and lateral film (Fig. 3) were named as ($X_{d1},Y_{d1A}$) and ($Z_{d1},Y_{d1L}$), and were equal to ($x_{d1}\times M_{d1A},y_{d1}\times M_{d1A}$) and ($z_{d1}\times M_{d1L},y_{d1}\times M_{d1L}$), respectively, where $M_{d1A}$ and $M_{d1L}$ were calculated through Eq. (A1) and represented as the magnification of dummy d1 on the AP and lateral film, respectively. Likewise, the location of dummy d3 on the AP and lateral film (Fig. 3) were described as ($X_{d3},Y_{d3A}$) and ($Z_{d3},Y_{d3L}$), and were equal to ($x_{d3}\times M_{d3A},y_{d3}\times M_{d3A}$) and ($z_{d3}\times M_{d3L},y_{d3}\times M_{d3L}$), respectively, where $M_{d3A}$ and $M_{d3L}$ were also calculated through Eq. (A1) and represented as the magnification of dummy d3 on the AP and lateral film, respectively. The coordinate of point O on the AP film and lateral film was described as ($X_{O},Y_{A\_O}$) and ($Z_{O},Y_{L\_O}$), respectively. On the AP film, the coordinate of G, ($X_{A1},Y_{A\_A1}$), and H, $X_{A2},Y_{A\_A2}$), can be calculated in order:

(14) $$X_{A1}=d_{13A}\times \text{sin}\alpha^{\prime }-2.8\times \text{cos}\alpha^{\prime }+X_{O}$$

(15) $$Y_{A\_A1}=d_{13A}\times \text{cos}\alpha^{\prime }+2.8\times \text{sin}\alpha^{\prime }+Y_{A\_O}$$

(16) $$X_{A2}=d_{13A}\times \text{sin}\alpha^{\prime }-2.8\times \text{cos}\alpha^{\prime }+X_{O}$$

(17) $$Y_{A\_A2}=d_{13A}\times \text{cos}\alpha^{\prime }+2.8\times \text{sin}\alpha^{\prime }+Y_{A\_O}$$

where $d_{12A}=\sqrt{{\left(X_{d1}-X_{d3}\right)}^{2}+{\left(Y_{d1A}-Y_{d3A}\right)}^{2}}$ and $\alpha^{\prime }$ was calculated by using Eq. (13). On the lateral film, the coordinate of the drawing G, ($Z_{A1},Y_{A\_L1}$), and H, ($Z_{A2},Y_{A\_L2}$), can be calculated as:

(18) $$Z_{A1}=Z_{A2}=d_{13L}\times \text{sin}\delta^{\prime }+Z_{O}$$

(19) $$Y_{L\_A1}=Y_{L\_A2}=d_{13L}\times \text{cos}\delta^{\prime }+Y_{L\_O}$$

where $d_{13L}=\sqrt{{\left(Z_{d1}-Z_{d3}\right)}^{2}+{\left(Y_{d1L}-Y_{d3L}\right)}^{2}}$ and $\delta^{\prime }$ was calculated by using Eq. (12).

Through (14), (15), (16), (17), (18), (19) and (A6), (A7), (A8), the coordinates of points G and H were reconstructed in 3D space and described as ($x_{G},y_{G},z_{G}$) and ($x_{H},y_{H},z_{H}$), respectively. Then, the location error $\Delta d_{A}$, the location difference between the practical and true coordinate of point A, can be written as:

(20) $$\Delta d_{A}=\left[\sqrt{{\left(x_{G}-x_{A1}\right)}^{2}+{\left(y_{G}-y_{A1}\right)}^{2}+{\left(z_{G}-z_{A1}\right)}^{2}}+\sqrt{{\left(x_{H}-x_{A2}\right)}^{2}+{\left(y_{H}-y_{A2}\right)}^{2}+{\left(z_{H}-z_{A2}\right)}^{2}}\right]/2$$

The location error on the x‐z plane, $\Delta d_{A_{\_\text{xz}}}$, for the purpose of dosimetric evaluation in the next section, can also be written as:

(21) $$\Delta d_{A\_xz}=\left[\sqrt{{\left(x_{G}-x_{A1}\right)}^{2}+{\left(z_{G}-z_{A1}\right)}^{2}}+\sqrt{{\left(x_{H}-x_{A2}\right)}^{2}+{\left(z_{H}-z_{A2}\right)}^{2}}\right]/2$$

Based on experience in clinical treatment, the α in this investigation was set at −20°,−10°, 0°, 10°, and 20°; the β was set at 10°, 20°, and 30°. The coordinate of point O was set at (R, R, R), where R is chosen from −10 to 10 cm with an interval of 2.5 cm. The $d_{1}$, the distance of dummy d1 away from point O, was set at 0.5 cm, 0.25 cm, 0 cm, −0.25 cm, and −0.5 cm, where the corresponding $d_{3}$ would be 2.5 cm, 2.25 cm, 2 cm, 1.75 cm, and 1.5 cm, respectively. For each chosen α,β, and $R,\Delta_{\text{dA}}$ was calculated for all the $d_{1}$‐$d_{3}$ pairs (0.5‐2.5, 0.25‐2.25, 0‐2, −0.25 1.75, and −0.5‐1.5), and its mean value and standard deviation were represented as $\bar{\Delta d_{A}}$ and $\sigma_{A}$, respectively. Likewise, the mean value of $\Delta d_{A_{\_\text{xz}}}$ for all the $d_{1}$‐$d_{3}$ pairs was represented as $\bar{\Delta d_{A_{\_\text{xz}}}}$.

## III. RESULTS & DISCUSSION

Table 1 demonstrates the mean differences $\bar{\Delta d_{A}}$ of all d^1^‐d^3^ pairs, where only the positive α and R were chosen to make the table look concise. The average and maximum $\sigma_{A}$ (standard deviations of $\Delta d_{A}$) calculated for all the α,β, and R, are 0.007 mm and 0.031 mm, respectively, which means the variation of the dummy location contributes trivially to the variation of $\bar{\Delta d_{A}}$. When α and R are fixed, generally speaking, $\bar{\Delta d_{A}}$ does not vary much with the changing of β; in addition, if β and R are fixed, $\bar{\Delta d_{A_{\_\text{xz}}}}$ does not vary much with the changing of α. Based on the general requirement of quality assurance, $\bar{\Delta d_{A}}$ should be within 2 mm, which will be achieved only when α is less than 10° with R equal to zero, or R is less than 5 cm with α equal to 0°. For an extreme case in which α is 20° with R equal to 10 cm, $\bar{\Delta d_{A}}$ would be greater than 6 mm. The mean difference is higher when α,β or R is higher, since F is further away from the isocenter, which would cause a higher error of magnification (Eq. (A1)) and minimization (Eq. (A4)) calculation.

**Table 1:** The mean differences $\bar{\Delta d_{A}}$ and $\bar{\Delta d_{A_{\_\text{xz}}}}$ (in mm) of all the $d_{1}$‐$d_{3}$ pairs. The coordinates of point O are in cm

*Point O Coordinate*	α	*0°*	*0°*	*0°*	*0°*	*10°*	*10°*	*20°*	*20°*	*20°*
(R,R,R)	β	*10°*	*20°*	*30°*	*10°*	*20°*	*30°*	*10°*	*20°*	*30°*
(0, 0, 0)	$\bar{\Delta d_{A}}$	0.22	0.27	0.33	1.94	1.94	1.94	3.61	3.61	3.62
(0, 0, 0)	$\bar{\Delta d_{A_{\_\text{xz}}}}$	0.10	0.19	0.28	0.10	0.19	0.28	0.10	0.20	0.28
(2.5, 2.5, 2.5)	$\bar{\Delta d_{A}}$	0.99	1.06	1.15	2.31	2.33	2.37	3.96	3.99	4.02
(2.5, 2.5, 2.5)	$\bar{\Delta d_{A_{\_\text{xz}}}}$	0.81	0.93	1.06	0.81	0.97	1.14	0.80	0.99	1.21
(5, 5, 5)	$\bar{\Delta d_{A}}$	1.85	1.94	2.07	2.85	2.90	3.00	4.38	4.45	4.57
(5, 5, 5)	$\bar{\Delta d_{A_{\_\text{xz}}}}$	1.51	1.68	1.87	1.52	1.75	2.04	1.48	1.80	2.18
(7.5, 7.5, 7.5)	$\bar{\Delta d_{A}}$	2.72	2.83	3.01	3.53	3.60	3.80	4.88	5.00	5.25
(7.5, 7.5, 7.5)	$\bar{\Delta d_{A_{\_\text{xz}}}}$	2.20	2.42	2.70	2.21	2.54	2.96	2.15	2.60	3.18
(10, 10, 10)	$\bar{\Delta d_{A}}$	3.59	3.73	3.97	4.32	4.43	4.72	5.46	5.65	6.06
(10, 10, 10)	$\bar{\Delta d_{A_{\_\text{xz}}}}$	2.88	3.16	3.54	2.90	3.32	3.89	2.82	3.41	4.20

According to the report “Recommendations from gynaecological (GYN) GEC ESTRO working group (II)” in 2006,^(27)^ the dose variation along the axis perpendicular to the intrauterine source at the level of point A can be approximately estimated as, with respect to the dose at point A, 10% increase per mm or 4% decrease per mm toward or away from the source, respectively. The dose gradient along the line that passes through the point A on the x‐y plane could be roughly taken as the same dose gradient along the axis described above. The location error of point A on the x‐z plane (Eq. (21)) was then used to estimate the dose error, according to the statement in the beginning of this paragraph. For R greater than 2.5 cm (Table 1), $\bar{\Delta d_{A_{\_\text{xz}}}}$ is greater than 1 mm, and the dose error would be higher than 4%. For an ordinary clinical case, if α,β, and R are equal to 10°, 30°, and 5 cm, respectively, in which point O is 8.7 cm away from the isocenter, $\bar{\Delta d_{A}}$ and $\bar{\Delta d_{A_{\_\text{xz}}}}$ will be 3.00 mm and 2.04 mm, respectively, and therefore, it is associated with a dose error higher than 8%. From the dosimetric point of view, the distance between the isocenter and the point O would be the main parameter that controls the dose error, since the tandem curvature β is not varied and the value of $\bar{\Delta d_{A_{\_\text{xz}}}}$ is nearly independent of α.

The reading errors of the gantry and collimator angle measured through the mechanical QA of our Toshiba DC50N simulator, which are 0.1° and −0.1°, respectively, will introduce a 1.12 mm reconstruction error^(34)^ (95% confidence interval). In this situation, all the mean differences shown in Table 1 should add 1.12 mm in quadrature for our facility. Therefore, for the above described clinical example, the location error of point A would be 3.20 mm in our clinic.

**Figure 4:**
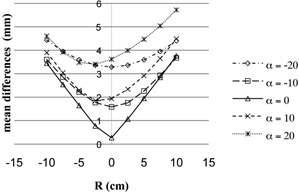
The illustration of the mean differences $\bar{\Delta d_{A}}$ averaged over all the βs (10°, 20°, and 30°), for α from −20° to 20° with an interval of 10° and R from −10 to 10 cm with an interval of 2.5 cm.

Since $\bar{\Delta d_{A}}$ did not vary much with the changing of β, to investigate the error regarding α and R, $\bar{\Delta d_{A}}$ was averaged over all the βs (10°, 20°, and 30°) and illustrated in Figure 4, for α from −20° to 20° with an interval of 10° and R from −10 to 10 cm with an interval of 2.5 cm. In Fig. 4, it is reasonable that the lowest point of the five curves appears where R is near zero, since there the F is close to the isocenter and the magnification of point A is close to 1.4 (point A was drawn 2.8 cm away from the tandem on AP film in Fig. 3); however, when carefully examining the curves α equal to 10° and 20°, the nadir points are near −2.5 cm, because there the F is even closer to the isocenter. When counting the calibration errors of the simulator and patient setup variations, it is really necessary to be careful to deliver the prescribed dose to the correct reconstructed point A and to avoid significant errors when treating cervical cancer patients.

## IV. CONCLUSIONS

It is essential to find the potential location errors of point A for cervical cancer radionuclide treatments in the clinic, since a slight variation in the location of point A can cause a significant dose variation. In this study, to obtain point A from orthogonal film reconstruction, the clinically used method is detailed through the geometric calculation, and the calculated result is compared with the true location. Physicists and physicians should be aware of the possible error and its related dependencies if treatment using the traditional procedure is performed in their facility. According to our calculation, if the tandem curvature angle is 30°, 10° rotated away from the gantry rotation axis (α=10°) in the AP view and the flange is 8.7 cm away from the isocenter (R=5 cm) with 0.1° and −0.1° reading errors of the simulator gantry angle and collimator angle, respectively, the location error of point A will be 3.2 mm. This error should be taken into consideration because point A is located at a high‐dose gradient area. Finally, to reduce the location error of point A when using the traditional method, it is necessary to move the couch or patient to make the mid‐point of the two points A near the isocenter (most importantly from the dosimetric point of view) and the tandem parallel to the gantry rotation axis as much as possible.

## ACKNOWLEDGMENTS

This work was supported in part by the National Science Council of Taiwan (NSC 101‐2221‐E‐214 ‐019).

## Supporting information

Supplementary Material

## Figures and Tables

**Figure A1:**
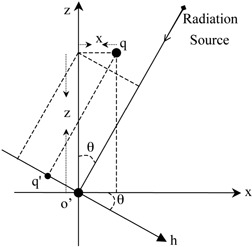
The magnification and projection (q′) on the h‐axis of ball point q (without consideration of any setup error) with its Cartesian coordinates (x, y, z). θ is the gantry angle rotated clockwise from the z‐axis and $o^{\prime }$ is the isocenter.

## Appendix A: Calculation Equations for the Two‐Film Reconstruction Technique

Using the same coordinate definition as in the Materials and Methods section, an object q in space could be reconstructed according to two films, with one shot at the gantry angle of zero degrees (AP direction) and the other at the gantry angle of θ degrees (Fig. (A1)). After digitizing the film into a treatment planning system, with the input gantry angle “θ” (relative to the gantry position of the “AP film”), the computer receives the information, M, $h_{M}$, and $y_{M}$, about the q point position on the film (Fig. (A1)), where M is the scale magnification, and $h_{M}$ and $y_{M}$ are the magnified coordinates of the q image on the h‐axis and y‐axis, respectively. With our back facing the gantry, the h‐axis and the y‐axis are the transverse axis of the crosshair image to the right, and the longitudinal axis of the crosshair image away from the gantry, respectively. For example, for the “AP film”, when θ=0°, the h‐axis is the same as the x‐axis; for the lateral film, when θ=‐90°, the h‐axis is the same as the z‐axis.

From this illustration, we could find that $\bar{{\text{qq}}^{\prime }}=\text{zcos}\theta +\text{xsin}\theta$ and the $q^{\prime }$ coordinate on the h‐axis is $–\text{zsin}\theta +\text{xcos}\theta .M,h_{M}$, and $y_{M}$ then could be written as:

(A1) M=SFD/[100−(z⋅cosθ+x⋅sinθ)]

(A2) $$h_{M}=(-z\cdot \text{sin}\theta +x\cdot \text{cos}\theta )\times M$$

(A3) $$y_{M}=y\times M$$

where *SFD* is the source–film distance. After reconstruction, the q point reconstructed from the coordinates, $\left(h_{M(\theta =\theta_{1})},y_{M(\theta =\theta 1)}\right)$ and $\left(h_{M(\theta =\theta_{2})},y_{M(\theta =\theta 2)}\right)$ of the two films with the gantry angle inputs, $\theta_{1}$ and $\theta_{2}$, respectively, has new coordinates, $\left(h_{n(\theta =\theta_{1})},y_{n(\theta =\theta 2)}\right)$, or the Cartesian coordinates, ($x_{n},y_{n},z_{n}$). Suppose that m is the scale minimization, we could then write

(A4) $$m=[100-(z_{n}\text{cos}\theta +x_{n}\text{sin}\theta )]/SFD$$

(A5) $$h_{n}=-z_{n}\text{sin}\theta +x_{n}\text{cos}\theta =h_{M}\times M$$

(A6) $$y_{n}=\frac{\left(y_{M(\theta =\theta_{1})}\times m_{\left(\theta =\theta_{1}\right)}+y_{M(\theta =\theta_{2})}\times m_{\left(\theta =\theta_{2}\right)}\right)}{2}$$

where $x_{n}$ and $z_{n}$ can be solved by Eq. (A5) with $\theta =\theta_{1}$ and $\theta =\theta_{2}$ to be

(A7) $$x_{n}=\frac{100h_{M(\theta =\theta_{1})}\cdot (h_{M(\theta =\theta_{2})}\cdot \text{cos}\theta_{2}-SFD\cdot \text{sin}\theta_{2})-100h_{M(\theta =\theta_{2})}\cdot (h_{M(\theta =\theta_{1})}\cdot \text{cos}\theta_{1}-SFD\cdot \text{sin}\theta_{1})}{\left(h_{M(\theta =\theta_{2})}\cdot \text{cos}\theta_{2}-SFD\cdot \text{sin}\theta_{2})(SFD\cdot \text{cos}\theta_{1}+h_{M(\theta =\theta_{1})}\cdot \text{sin}\theta_{1})-(h_{M(\theta =\theta_{1})}\cdot \text{cos}\theta_{1}-SFD\cdot \text{sin}\theta_{1})(SFD\cdot \text{cos}\theta_{2}+h_{M(\theta =\theta_{2})}\cdot \text{sin}\theta_{2}\right)}$$

(A8) $$z_{n}=\frac{100h_{M(\theta =\theta_{2})}-(SFD\cdot \text{cos}\theta_{2}+h_{M(\theta =\theta_{2})}\cdot \text{sin}\theta_{2})\cdot x_{n}}{h_{M(\theta =\theta_{2})}\cdot \text{cos}\theta_{2}-SFD\cdot \text{sin}\theta_{2}}$$

where $\theta_{1}$ and $\theta_{2}$ can be exchanged with each other in (A7), (A8) and lead to the same results.
