# Supplementary material for: Geometric error of cervical point A calculated through traditional reconstruction procedures for brachytherapy treatment
Source: J Appl Clin Med Phys. 2015 Sep 8;16(5):457–68. doi: 10.1120/jacmp.v16i5.5558 (PMC5690162; doi:10.1120/jacmp.v16i5.5558)
Supplement: Supplementary file 1 — Supplementary Material [file ACM2-16-457-s001.docx]

**Geometric Error of Cervical Point A Calculated through Traditional Reconstruction Procedures for Brachytherapy Treatment**

Liyun Chang^1a^*,* Sheng-Yow Ho^2^, Shyh-An Yeh^1,3^, Tsair-Fwu Lee^4^ and Pang-Yu Chen^5b^

*Department of Medical Imaging and Radiological Sciences^1^, I-Shou University, Kaohsiung, Taiwan; Department of Radiation Oncology^2^, Chi Mei Medical Center, Liouying, Tainan, Taiwan; Department of Radiation Oncology^3^, E-Da Hospital, Kaohsiung, Taiwan; Medical Physics and Informatics Laboratory^4^, Department of Electronics Engineering, National Kaohsiung University of Applied Sciences, Kaohsiung, Taiwan; Department of Radiation Oncology^5^, Sinlau Christian Hospital, Tainan, Taiwan*

^a^ Corresponding author: Liyun Chang, Department of Medical Imaging and Radiological Sciences, I-Shou University, Kaohsiung, Taiwan. Email: [liyunc@isu.edu.tw](mailto:liyunc@isu.edu.tw)

^b^ Pang-Yu Chen, Department of Radiation Oncology, Sinlau Christian Hospital, Tainan, Taiwan. Email: [pangyuchen@yahoo.com.tw](mailto:pangyuchen@yahoo.com.tw)

Running title: Geometric Error of Point A
